# The Role of *Clostridioides difficile* Within the One Health Framework: A Review

**DOI:** 10.3390/microorganisms13020429

**Published:** 2025-02-16

**Authors:** Sotiris Alexiou, Anastasia Diakou, Melania Kachrimanidou

**Affiliations:** 1 Department of Microbiology, Medical School, Aristotle University of Thessaloniki, 54124 Thessaloniki, Greece; sotirisalexiou.s.a@gmail.com; 2 Laboratory of Parasitology and Parasitic Diseases, School of Veterinary Medicine, Faculty of Health Sciences, Aristotle University of Thessaloniki, 54124 Thessaloniki, Greece; diakou@vet.auth.gr

**Keywords:** *Clostridioides difficile*, One Health, human, animals, environment, food

## Abstract

*Clostridioides difficile* is the leading cause of antibiotic-associated diarrhea in hospitalized patients. In recent years, the incidence of *C. difficile* infection (CDI) has increased globally, with a notable rise in community-associated CDI (CA-CDI). The presence of the microorganism in animals, the environment, and food suggests that these sources may contribute to the spread of the infection in the community. This review applies a One Health approach, integrating human, animal, and environmental health, to provide a comprehensive strategy for understanding and managing this pathogen. Findings reveal the widespread dissemination of *C. difficile* in animals, the environment, and food. The predominant PCR ribotypes identified were RTs 078 and 014/020, followed by RTs 126, 001, 002, 009, 010, and 033. *C. difficile* strains exhibited resistance to multiple antimicrobial agents, including clindamycin, erythromycin, fluoroquinolones, cephalosporins, and tetracyclines. Discriminative typing methods, such as whole-genome sequencing, revealed clonal relationships between *C. difficile* strains from humans and animals, indicating either direct transmission or a common environmental source of infection. The high genetic similarity between isolates from the environment and humans indicates potential environmental contamination. Additionally, clusters of *C. difficile* strains found in food and humans indicate a possible foodborne transmission route. This review summarizes the current knowledge on the role of *Clostridioides difficile* within the One Health framework.

## 1. Introduction

*Clostridioides difficile* (formerly *Clostridium difficile*) is a Gram-positive, spore-forming, anaerobic bacterium that colonizes the gastrointestinal tract of humans and animals and is widely spread in the environment [1,2]. It is the primary cause of infectious diarrhea in hospitalized patients, with broad-spectrum antibiotics being the most significant risk factor for *C. difficile* infection (CDI), as they disrupt gut microbiota, allowing for spore germination and toxin production [3,4]. The pathogenesis of this microorganism is primarily mediated by two exotoxins, toxin A and toxin B, encoded in the pathogenicity locus (PaLoc) of the *C. difficile* genome [5]. Some *C. difficile* strains produce a third toxin, binary toxin (CDT), which is frequently observed in strains associated with the increased severity of *C. difficile* infection [6].

*C. difficile* infection can present with various clinical manifestations, ranging from mild diarrhea to severe and potentially fatal conditions, such as pseudomembranous colitis and toxic megacolon [7]. Historically, CDI was considered primarily a healthcare-associated infection (HA-CDI). HA-CDI is defined as CDI with symptom onset at least 48 h after hospital admission or within four weeks after hospital discharge [8]. In recent decades, the frequency, severity, recurrence rates, and mortality associated with CDI have significantly increased [9]. CDC’s most recent surveillance data report the crude overall incidence rate to be 116.1 cases per 100,000 persons, with a higher incidence of community associated cases (62.1 cases per 100,000 persons) compared with healthcare-associated cases (54.0 cases per 100,000) [10]. Alongside this, significant shifts in the global epidemiology of the disease have been observed, with the prevalence of community-associated CDI (CA-CDI) rising substantially [11]. CA-CDI is defined as a case of CDI with the onset of symptoms outside of healthcare facilities or within 48 h of admission to hospital or more than 12 weeks after [12]. CA-CDI accounts for approximately 33% of all CDI cases in Europe [13], with notable variation between countries. For instance, in Ireland, 38.4% of CDI cases are community-associated, while in the Netherlands, the proportion is 42.7%, and in Slovakia, it is 22.3%. Similarly, in Australia, 26% of CDI cases are community-associated [14]; whereas, in the USA, the percentage rises to 51% [15].

Investigations into potential community reservoirs for the pathogen reveal that *C. difficile* is widespread in animals, the environment, and food [16,17]. Specifically, the bacterium has been isolated from various animal species, including livestock, companion animals, horses, and wildlife [18]. Furthermore, it is found in diverse environments, such as soil, water, sewage, and various surfaces, and has also been detected in foods of both animal and plant origin [19].

**Figure 1:**
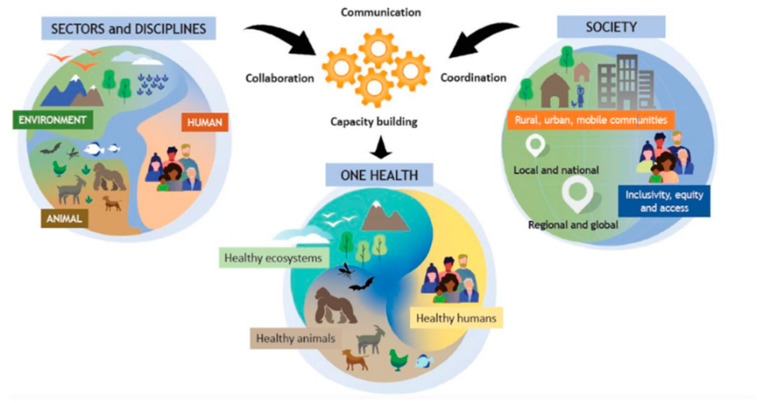
The One Health concept as developed by the One Health High-Level Expert Panel (OHHLEP), illustrating the interconnectedness of the environment, animals, humans, and society. Communication, collaboration, and coordination among different scientific disciplines are essential to support healthy ecosystems, humans, and animals. Available under the Creative Commons CC0 1.0 license [21].

The detection of identical *C. difficile* strains in humans, animals, food, and the environment suggests zoonotic transmission, with the environment and food acting as connecting links [20]. These findings underscore the need to study *C. difficile* within a One Health framework. The World Health Organization (WHO) defines One Health as an integrated, unifying approach that aims to sustainably balance and optimize the health of people, animals, and ecosystems, as illustrated in Figure 1 [21]. This approach fosters collaboration among various scientific fields, including medicine, veterinary science, biology, environmental science, and public health to address global issues, such as emerging infectious diseases, antimicrobial resistance, and food safety. For instance, Knight et al. applied the One Health concept in their study of *C. difficile* sequence type 11 (ST11) and 258 (a closely related lineage), analyzing isolates from humans, animals, and environmental sources across multiple continents [20]. Their findings emphasized zoonotic and anthroponotic transmission, highlighting the importance of an integrated approach to understanding and managing *C. difficile* spread and antimicrobial resistance.

The purpose of this study is to review the literature on the role of *Clostridioides difficile* within the One Health framework, with a particular focus on potential transmission events.

## 2. *Clostridioides difficile* in Animals

*C. difficile* has been isolated from a variety of animal species, including food-producing animals, household pets, and wildlife. The prevalence and main PCR ribotypes of *C. difficile* detected in these animals are summarized in Table 1.

### 2.1. Clostridioides difficile in Food-Producing Animals: Pigs

*C. difficile* has been extensively studied in pigs and is recognized as a significant pathogen, causing diarrhea primarily in piglets [22]. The detection of toxigenic strains in both healthy and sick pigs highlights the role of asymptomatic carriers as reservoirs of this pathogen [60]. Furthermore, the isolation of multiple *C. difficile* strains known to cause disease in humans suggests that neonatal pigs may serve as a potential reservoir for human *C. difficile* infections. PCR ribotype 078 was the predominant type identified in several studies [22,23,24,25,26], with RTs 014, 126, 033, 038, and 046 also frequently observed [20,27,28,29,30]. Some strains of *C. difficile* demonstrated antimicrobial resistance particularly to fluoroquinolones, cephalosporins, clindamycin, erythromycin, and tetracyclines [27,31]. Τhe most common PCR ribotypes have frequently been reported as resistant to some of these antibiotics; however, in some studies, the correlation between PCR ribotypes and resistance was either not investigated or not explicitly mentioned.

Dingle et al. found that 85% of the strains isolated from humans and pigs were genetically related, regardless of geographic location [61]. The use of tetracyclines in agriculture and animal farming has led to selective pressure, contributing to the evolution of tetracycline-resistant *C. difficile* RT078. The study also suggested that humans may acquire RT078 through the food chain or environmental exposure.

The whole-genome sequencing of forty *C. difficile* RT014 strains from pigs and humans in Australia revealed that twelve human isolates were genetically identical (≤2 SNVs) to six pig isolates [27]. These isolates were distributed across a large geographic area (~800 km), including both urban and rural areas, and over a long period of time (11–12 months), with 50% of human cases occurring without recent hospital exposure. Among human-derived *C. difficile* RT014 strains, non-susceptibility was observed to clindamycin, erythromycin, and ceftriaxone; whereas, pig-derived strains exhibited non-susceptibility to these antibiotics as well as to tetracycline [27]. These findings indicate a persistent community reservoir and interspecies transmission events. Although evidence of long-range interspecies transmission exists, the exact mode of transmission between pigs and humans remains unclear. *Clostridium difficile* infection (CDI) is a complex issue, and understanding the dynamics of CDI transmission, particularly in relation to the food chain and community environments, is still in its early stages. The continuous monitoring of *C. difficile* strains at both the molecular and phenotypic levels in humans, animals, food, and the environment is essential for identifying potential intervention points and reducing the overall burden of CDI.

A European study collected *C. difficile* RT078 strains from HA-CDI, CA-CDI, asymptomatic farmers, and pigs, revealing genetically related isolates (≤2 SNPs), primarily within countries and among different reservoirs [62]. In Dutch farms, isolates of RT078 from asymptomatic farmers and pigs were genetically identical (≤2 SNVs, ANI ≥99.73), forming clonal clusters and suggesting interspecies transmission [20,37].

### 2.2. Clostridioides difficile in Food-Producing Animals: Cattle

*C. difficile* has been isolated from both healthy cattle and cattle with diarrhea, with enteritis of preweaning neonatal calves being the most common CDI manifestation in this species [43]. The asymptomatic colonization of the gut by *C. difficile* is possible in healthy cattle, but the disruption of the gut microbiota, such as after antibiotic treatment, can lead to infection and diarrhea. Τhe detection of *C. difficile* has been associated with calves, antibiotic administration, and specific breeds, such as Limousin and Holstein [33,34,35,63]. The most frequently detected PCR ribotypes were 033, 126, 078, and 014/020 [2,26,30,34,35]. Many strains exhibited resistance to antibiotics, such as erythromycin, clindamycin, moxifloxacin, and tetracyclines [34,35,64]. For instance, Bandelj et al. reported high antimicrobial resistance in RT012 isolates, particularly against erythromycin, clindamycin, and rifampicin, while resistance to tetracycline was also observed [64]. Likewise, Masarikova et al. identified certain RT033 strains as multidrug-resistant, exhibiting resistance to ciprofloxacin, tetracycline, clindamycin, and moxifloxacin [34].

Some studies have reported identical strains of *C. difficile* in cattle and humans. An Italian study using the phylogenetic analysis of genomic DNA fingerprinting patterns from agarose-based PCR ribotyping found a 92% similarity between *C. difficile* RT078 strains from cattle and humans with CA-CDI [24]. In Australia, clonal groups (≤2 SNVs) were identified among *C. difficile* RTs 126, 127, and 033/288 from calf feces, carcasses, and both HA-CDI and CA-CDI cases [20]. Notably, an RT078 strain from cattle in Canada clustered with isolates from humans in the United Kingdom (ANI ≥ 99.75), suggesting the clonal spread of the pathogen across geographic boundaries [37]. This global dissemination could be facilitated by human movement, the international trade of animals and animal products, as well as by vectors, such as migratory birds.

### 2.3. Clostridioides difficile in Food-Producing Animals: Poultry

Data on *C. difficile* infection in poultry are limited, with cases reported in both asymptomatic animals and those presenting clinical disease. Frequently detected PCR ribotypes included 001, 002, 014/020 [26,38], and 078 [37,39], many of which are also found in humans. Berger et al. applied whole-genome sequencing and MLVA analysis, showing that RT025 isolates from humans, chickens, and soil samples, as well as RT084 isolates from humans and chickens, were genetically related [40]. The detected clusters for RT025 and RT084 among human, chicken, and soil isolates suggest a possible epidemiological connection, which could highlight a potential ongoing transmission between humans and environmental sources and may indicate zoonotic potential. Frentrup et al. reported a close phylogenetic relationship (≤2 SNPs) between *C. difficile* RT001 isolates from CDI patients, chicken manure, and chicken meat [38].

### 2.4. Clostridioides difficile in Food-Producing Animals: Goats and Sheep

Goats and sheep have been characterized primarily as asymptomatic carriers of *C. difficile* [16,43]. In sheep, RTs 126 and 078 were detected [24,41], while in goats, RTs 010, 014/020, 045, and 110 were identified [26,44]. Most PCR ribotypes isolated from small ruminants have also been detected in humans. Genetic analyses identified similarities between *C. difficile* RT078 strains from CA-CDI cases and sheep, suggesting interspecies transmission or shared environmental contamination [24].

### 2.5. Clostridioides difficile in Horses

*C. difficile* causes diarrhea in both foals and adult horses [16]. Frequently detected PCR ribotypes were RTs 033, 009, 010, 012 [45,46], 014/020 [30,46], 078 [26,37], 126 [26], and 127 [20]. In South Korea, *C. difficile* strains isolated from horse feces belonged to PCR ribotypes RT078, RT012, RT009, and RT010, exhibiting multidrug resistance to antibiotics such as cefotaxime, clindamycin, erythromycin, gentamycin, penicillin, and tetracycline [47]. Additionally, RT078 strains were also resistant to moxifloxacin, while RT012 and RT009 showed resistance to rifampicin. Higher isolation rates of the pathogen were associated with the age of the animals (foals under one year), antibiotic treatment, diarrhea, and the spring and summer seasons. A study in Australia conducted the whole-genome sequencing of RT012 strains from humans and horses, presenting further support the hypothesis that horses represent a possible reservoir for *C. difficile* dispersal to humans [46]. The SNP analysis identified three horse strains and one human strain differing by two SNPs, suggesting interspecies transmission or exposure to a common environmental source.

### 2.6. Clostridioides difficile in Household Pets: Dogs and Cats

The role of *C. difficile* in cases of the intestinal disease of companion animals remains unclear. Although infections in dogs have been documented [30], some studies reveal that dogs with diarrhea containing toxigenic *C. difficile* strains do not exhibit the typical macroscopic or microscopic lesions in the colon, associated with CDI [48]. Dogs frequently act as asymptomatic carriers of toxigenic and antimicrobial-resistant *C. difficile* strains. Resistance has been noted in clindamycin, erythromycin, moxifloxacin, tetracyclines, and occasionally, metronidazole [48,49,65,66]. Data on cats are limited, with the pathogen isolated from both healthy cats and those with diarrhea.

Beyond fecal samples, the pathogen has also been detected in other biological materials from dogs, such as nasal swabs, bronchoalveolar lavage fluid [67], and paw swabs [55,68]. While the potential significance of these findings for transmission routes remains unclear, the presence of *C. difficile* in these biological materials could represent a possible source of transmission, suggesting the need for further investigation into alternative modes of spread. The most common reported RTs in dogs included 014/020, 106, 010, 012, 078, and 001 [26,30,39,48,50]. Similarly, cats most frequently harbor RTs 010, 009, 014/020, 106, and 001 [49,52,53].

Close genetic relationships between *C. difficile* strains in companion animals and humans have been identified. Specifically, identical *C. difficile* ST8 strains (SNP = 1) were observed in a household dog and its owner with CDI [54]. In Denmark, cgMLST analysis revealed similarities (2–5 allelic differences) between strains isolated from dog feces and human clinical case strains, specifically belonging to RTs 014/020, 106/174, and two non-toxigenic strains (ST26) [50]. Finally, in Portugal, clusters of genetically identical RT 106 strains (SNPs ≤ 2) were identified, isolated from humans, dogs, and cats [49]. These findings indicate possible interspecies transmission or shared environmental contamination.

### 2.7. Clostridioides difficile in Wildlife

*C. difficile* has also been isolated from various wildlife species. In Canada, urban rats (*Rattus norvegicus*, *Rattus rattus*) were found to carry multiple PCR ribotypes, including 001, 078, 014, and 027 [56]. In the Netherlands, wild rodents (mice and rat species) and insectivores (shrew species) trapped in pig and cattle farms carried PCR ribotypes 005, 010, 014, 015, 078, and 087, which are associated with human CDI [57]. In Canada, mammals such as striped skunks (*Mephitis mephitis*) and Virginia opossums (*Didelphis virginiana*), trapped in pig farms and protected areas, were found to harbor toxigenic *C. difficile* strains in their feces, including RTs 078 and 002 [58]. Finally, a close phylogenetic relationship (≤2 SNVs) has been observed between *C. difficile* RT126 isolates from kangaroo feces and two human CDI cases in Australia [20].

## 3. *Clostridioides difficile* in the Environment

*C. difficile* has been isolated from soil [41,69], water [70,71], air [29], sewage [72,73], manure [64,74], compost [75,76], and various surfaces [55,77]. A wide range of PCR ribotypes has been identified, many of which are associated with human CDI. Notable RTs include 078 [23,37], 014/020 [35,64], 001, 002 [38], 126 [35,78], 046 [28,79], and 033 [35,64]. Prevalence rates vary significantly, from 0% in drinking water samples to as high as 100% in sewage samples [41]. The prevalence and main PCR ribotypes of *C. difficile* detected in various environmental sources are summarized in Table 2.

### 3.1. Clostridioides difficile in the Natural Environment

In the USA, Williamson et al. isolated the pathogen from water and soil samples [85]. SNP analysis revealed genomic overlap between isolates from dogs and soils (ST3-NT, ST15), as well as between environmental isolates (soil and water) and human clinical strains (ST42, ST3-T). Similarly, in Germany, strains isolated from lake sediment and wastewater inflows were closely related to human-derived strains (5–50 SNPs) [86]. These findings underscore the potential for transmission of the pathogen between humans and the environment.

### 3.2. Clostridioides difficile in the Livestock Farming Environment

A substantial number of environmental samples have been collected from livestock facilities. Alves et al. isolated *C. difficile* in pig farms from soil, manure, air, and wastewater samples, highlighting the potential for the airborne transmission of spores within farming facilities [29,41]. This suggests that airborne spores could contribute to the spread of *C. difficile* within farming environments, potentially affecting both animals and farm workers. Such transmission routes increase the risk of human exposure and cross-species transmission, which is significant for public health monitoring. Core genome SNV analysis revealed high genetic similarity between RT033-variant isolates from pigs and their surrounding environment. Additionally, the pathogen was isolated from samples of composted pig manure, including the hypervirulent RT078 strain, indicating that the use of composted manure on agriculture could facilitate the spread of the pathogen through the food chain [75]. In Sweden, RT046 was isolated from pigs, personnel clothing, soil, and stream water [28,79]. The stream strain was grouped with a human strain (≤2 cgMLST allelic differences) recovered three years earlier in a different geographic area. Most environmental and pig-derived strains were highly similar (≤6 cgMLST allelic differences), though they diverged from human strains. These findings suggest a potential link in the transmission of *C. difficile* between animals, humans, and the environment, highlighting the importance of tracking the pathogen across different reservoirs.

### 3.3. Clostridioides difficile in the Veterinary Clinic Environment

The environment of veterinary clinics has been identified as a potential reservoir for *C. difficile*. Villagomez-Estrada et al. reported that 4% of surface samples in a veterinary hospital in Spain tested positive for the pathogen, with PCR ribotypes 014 and 078 identified [80]. These strains were resistant to multiple antibiotics, including metronidazole, reflecting the extensive use of antibiotics in veterinary medicine. At a veterinary school in Poland, high contamination rates (96%) of *C. difficile* spores were observed on the soles of shoes of veterinarians, students, and support staff [81].

### 3.4. Clostridioides difficile in the Urban Environment

Urban environments also harbor *C. difficile*. In Spain, recreational sandboxes for children and dogs were found to be contaminated, with rates of 45% and 60%, respectively [82]. RTs 014, 009, and 039 were present in both types of samples; however, AFLP analysis indicated that these were distinct strains. In Australia, toxigenic *C. difficile* strains were detected in 58.5% of lawn samples from public areas [83]. The contamination of the lawn was likely related to the application of animal manure or human biosolids as fertilizer. Similarly, from the immediate outdoor environment of six hospitals in Australia, *C. difficile* was isolated from 60.4% of samples consisting of soil, sand, lawn, mulch, and mixed samples [87]. Toxigenic strains, which cause CDI in humans, were isolated, including RTs 014/020, 103, 056, 106, and 017.

### 3.5. Clostridioides difficile in the Household Environment

In Slovenia, identical PCR ribotypes were isolated from the slippers, shoes, and paw pads of dogs in three households. SNV analysis showed that RT014/020 strains were clonal within each household, suggesting a common source of contamination [68]. In Australia, *C. difficile* was recovered from manure, soil, compost, and shoe soles in 95.6% (22/23) of surveyed home gardens, with an overall isolation rate of 67% across all samples [74]. The widespread presence of spores was linked to the use of contaminated animal manure and compost as fertilizers. In this context, sodium hypochlorite at an alkaline pH has been shown to be effective in eliminating *C. difficile* spores [88].

### 3.6. Clostridioides difficile in Wastewater Treatment Plants

Wastewater treatment plants are significant sources of environmental contamination with *C. difficile*. Moradigaravand et al. highlighted the release of toxigenic *C. difficile* strains into surface waters, including lakes, rivers, and coastal areas, from eighteen wastewater treatment plants, ten of which received hospital wastewater [89]. SNP analysis revealed high similarity (≤2 SNPs) between clinical CDI strains and those isolated from wastewater. In Australia, *C. difficile* was found in twelve wastewater treatment plants, with prevalence rates ranging from 40% in irrigation water to 94.3% in biosolids [72]. PCR ribotypes associated with human CDI, such as RTs 014/020, 078, and 126, were commonly identified. Additionally, in Italy, MLVA analysis found identical *C. difficile* isolates in humans (RTs 011/018, 126), sewage (RTs 011/018, 126), and raw milk (RT011/018, new RT) (STRD ≤ 2) [2]. Similarly, Knight et al. reported the genetic clustering (≤2 SNVs) of *C. difficile* RT033 strains isolated from piglet feces, soil irrigated with wastewater effluent, and treated wastewater [20].

## 4. *Clostridioides difficile* in Foods

The presence of *C. difficile* has been investigated in various food types, including animal-derived foods (meat, seafood, dairy) and plant-derived foods (vegetables and grains). The prevalence and main PCR ribotypes of *C. difficile* detected in food are summarized in Table 3.

### 4.1. Clostridioides difficile in Animal-Derived Foods: Meat

Studies on *C. difficile* in meat have identified its presence in products such as pork, beef, and chicken, with the most frequently isolated PCR ribotypes being 078, 014/020, and 001 [90,91,92].

In an Australian study, the prevalence of the pathogen in calves younger than fourteen days was 25.3%, indicating that young slaughter age was a significant factor [36]. In a three-year surveillance study conducted in Slovenia, the pathogen was isolated from 3.4% of raw poultry, pork, and beef samples [90]. The detected RTs 001, 078, and 014/020 have also been isolated in CDI patients, animals, and the environment in Slovenia, highlighting the risk of foodborne transmission.

Rahimi et al. studied the pathogen in various types of raw meat, reporting the highest prevalence in buffalo and goat meat, while camel meat was negative for the pathogen [92]. The isolated strains belonged to RT078 and exhibited resistance to clindamycin, erythromycin, tetracyclines, ciprofloxacin, and gentamicin.

In Canada, *C. difficile* was isolated from frozen pork and beef samples, indicating that the pathogen’s spores can survive freezing [98]. In contrast, Pires et al. found no evidence of *C. difficile* in ready-to-eat meat samples, including beef, pork, chicken, and hamburgers [99].

### 4.2. Clostridioides difficile in Animal-Derived Foods: Seafood

Two studies in Italy examined the contamination of mussels and clams with *C. difficile*. Agnoletti et al. reported the pathogen in 11.6% of mussel and 23.2% of clam samples [93], while Troiano et al. identified it in 3.6% of mussels and 23.1% of clams [94]. The presence of the pathogen in seafood was not associated with bacterial indicators of fecal contamination in the water (e.g., *Escherichia coli* and *Salmonella* spp.) but reflected its widespread environmental presence. The most prevalent RTs from both studies were 014, 078, 126, 002, 010, and 018, many of which are frequently implicated in human CDI cases. Additionally, antimicrobial resistance was observed, particularly to erythromycin, clindamycin, and fluoroquinolones, with one strain exhibiting resistance to vancomycin. These findings suggest that bivalve mollusks could serve as a potential source of human infection.

### 4.3. Clostridioides difficile in Animal-Derived Foods: Dairy

*C. difficile* was recovered from raw bovine milk samples [2]. One strain belonged to a new RT, while others were identified as RTs 011/018 and 078. MLVA analysis revealed high genetic similarity between *C. difficile* RT011/018 strains from humans, raw bovine milk, and treated wastewater (STRD ≤ 1).

### 4.4. Clostridioides difficile in Plant-Derived Foods

The presence of *C. difficile* in plant-derived foods has mainly been studied in vegetables and grains. In a U.S. hospital, the pathogen was detected in a vegetable bread sample [100]. The strain belonged to RT027, and the positivity rate in vegetables was 0.1%. In three Italian hospitals, *C. difficile* was recovered from 1.9% of vegetable samples, with a positive sample from lettuce belonging to RT126 [95].

Tkalec et al. isolated *C. difficile* in 6.1% of raw vegetables in Slovenia [90]. Positive samples were found in lettuce, ready-to-eat salads, and root vegetables, with RTs 001, 012, and 010 identified. A European study investigating the prevalence of *C. difficile* in potato samples found a positivity rate of 22.4% across countries [96]. A statistically significant difference in *C. difficile* isolation was observed between soil-covered potatoes and visibly clean ones. The most prevalent PCR ribotypes were 078/126, 014/020, 010, and 023, which are frequently isolated from humans, animals, and the environment. Additionally, RT033 was isolated from a potato sample in Australia [20]. These findings suggest that potatoes could pose a public health risk. Finally, MLVA analysis grouped a human RT126 strain with an RT078 strain from a ready-to-eat salad (STRD = 6) [2].

## 5. Transmission Cycle of *Clostridioides difficile*

*C. difficile* is a well-established pathogen in healthcare settings and the leading cause of hospital-acquired diarrhea [101]. Within hospitals, the transmission of the pathogen occurs through direct contact with colonized patients or via contact with contaminated surfaces, materials, instruments, or medical personnel [102]. Recent studies have highlighted the role of asymptomatic carriers in hospital transmission, as patients arrive already colonized with the pathogen [103]. The continuous introduction of new *C. difficile* strains into hospitals underscores the critical role of community reservoirs, including animals, food, and the environment, in the transmission cycle. Clonal relationships observed among certain *C. difficile* strains indicate potential transmission between humans and animals or exposure to common environmental sources. Specifically, the direct transmission of the pathogen between humans and animals is likely in shared environments, such as households, farms, animal shelters, veterinary hospitals, zoos, and other settings where humans and animals coexist. However, direct transmission is rarely reported. For instance, Knetsch et al. demonstrated that asymptomatic farmers and pigs in Dutch swine farms were colonized with identical (SNP = 0) or nearly identical (≤2 SNPs) *C. difficile* RT078 strains, suggesting possible transmission between them [104]. Similarly, while direct transmission between companion animals and their owners has not been conclusively proven, a case study involved genetically identical *C. difficile* ST110 strains (related to RT020) in a 10-month-old infant and a dog, both with diarrhea [105].

Beyond direct fecal–oral transmission, *C. difficile* can spread over large geographic distances, via environmental and foodborne routes. Livestock colonized with *C. difficile* can contaminate meat during slaughter through the leakage of intestinal contents onto the carcass. Meat contamination can also occur during transport, storage, and processing [16]. The pathogen’s spores can survive recommended cooking temperatures (71 °C) for over two hours [106] and remain viable under refrigeration or freezing conditions [107]. Additionally, animal-derived foods, fruits, vegetables, and grains can become contaminated with *C. difficile* through certain agricultural practices. The application of fertilizers derived from animal manure, compost products, or human biosolids can introduce *C. difficile* to crops [72,75]. Additionally, the use of treated wastewater for agricultural irrigation can disperse *C. difficile* spores onto crops [72]. Treated wastewater flowing into surface waters can contaminate lakes, seas, and rivers, as well as fish and edible bivalves living in these aquatic ecosystems [89]. Currently, there are no documented cases of CDI resulting from the consumption of contaminated food. Therefore, *C. difficile* is considered an unspecified foodborne agent, and further research is needed to evaluate the viability of its spores and the growth potential of the microorganism in foods [108].

Other transmission routes, such as airborne dissemination or vectors like birds, rodents, and arthropods, can spread the pathogen across large geographic areas [29,109]. As previously discussed, airborne spores collected from swine units tested positive for *C. difficile*, supporting the possibility of airborne transmission, while vermin in swine units were found to carry *C. difficile* PCR ribotypes 078 and 045, suggesting a role in the spread of the pathogen.

Finally, the natural environment has been identified as a natural reservoir for the microorganism, carrying highly divergent strains capable of infecting humans and animals, who, in turn, excrete *C. difficile* spores through their feces, thus perpetuating the transmission cycle [71].

## 6. Conclusions

This review confirms that *Clostridioides difficile* is a critical pathogen within the One Health framework. The application of high-resolution microbial genomics, integrating data from clinical, veterinary, and environmental sources, serves as an ideal model for advancing the understanding of epidemiological and genetic factors contributing to the emergence, evolution, and spread of CDI [110].

Current strategies for controlling *C. difficile* primarily focus on antibiotic stewardship and infection control policies within healthcare settings [111]. Reducing antibiotic use in agriculture and livestock is also critical. In this context, administering non-toxigenic *C. difficile* strains, such as strain Z31, has shown promise in reducing CDI incidence in piglets [112].

Mitigating environmental contamination is another priority. Vaccine development aimed at reducing colonization and infection in both animals and humans is under consideration [113]. Composting biosolids and the anaerobic digestion of sewage sludge have been effective in reducing *C. difficile* levels, though the complete elimination of the pathogen remains challenging [17]. Additionally, heating foods to temperatures above 85 °C is recommended to prevent ingestion of *C. difficile* spores through contaminated food [114]. Enhancing host resistance to CDI by modulating the gut microbiome also represents a promising therapeutic approach [17,43].

Finally, standardizing culture and PCR ribotyping methods for this microorganism, establishing a comprehensive PCR ribotype library, and adopting a unified ISO procedure for isolating *C. difficile* from food products are essential steps for conducting effective global surveillance studies on this pathogen [108,115].

## Figures and Tables

**Table 1:** Presence of *C. difficile* in animals.

Animal Species	Prevalence	Main PCR-Ribotypes	Citation
Pigs	0.3–92%	078, 014, 126, 033, 038, 046	[20,22,23,24,25,26,27,28,29,30,31,32]
Cattle	0–60%	033, 126, 078, 014/020, 127	[2,20,26,30,33,34,35,36]
Poultry	0–15.4%	001, 002, 014/020, 078, 025, 084	[26,37,38,39,40,41,42]
Goats, sheep	0.6–10.1%	126, 078, 010, 014/020, 045, 110	[16,24,26,41,43,44]
Horses	2.4–31.8%	033, 009, 010, 014/020, 078, 126, 127	[20,26,30,37,45,46,47]
Dogs	2.1–26%	014/020, 106, 010, 078, 012, 001	[26,30,39,48,49,50,51]
Cats	0–16.4%	010, 009, 014/020, 106, 001	[49,52,53,54]
Wildlife	3.5–39.2%	078, 014/020, 002, 009, 010, 027, 005, 015, 087	[44,55,56,57,58,59]

**Table 2:** Presence of *C. difficile* in the environment.

Environment	Prevalence	Main PCR-Ribotypes	Citation
Natural	14.4–47.3%	014/020, 010, 106	[69,70,71]
Livestock Farming	0–91.3%	078, 014/020, 126, 001, 002, 033, 046	[23,32,38,41,64,75,79]
Veterinary clinic	4–96%	014/020, 078, 010, 009	[80,81]
Urban	22–60%	014/020, 078, 002, 010, 009, 039	[55,82,83]
Household	18.9–83.3%	014/020, 001, 002, 010	[68,74,77,84]
Wastewater Treatment plants	40–100%	078, 126, 014/020, 033, 127, 001	[20,41,72,73,78]

**Table 3:** Presence of *C. difficile* in foods.

Food	Prevalence	Main PCR-Ribotypes	Citation
Meat	0–25.3%	078, 014/020, 001	[33,36,90,91,92]
Seafood	3.6–23.2%	014, 078, 126, 002, 010, 018	[93,94]
Dairy	-	011/018, 078	[2]
Plant-derived (vegetables, grains)	0–22.4%	078, 014/020, 001, 126, 027	[2,95,96,97]

(-) Data not available.

## Data Availability

No new data were created or analyzed in this study. Data sharing is not applicable to this article.
